# Single Stage Reconstruction of Large Calvarial Exposure after Tumor Resection: A 3-Year Experience

**DOI:** 10.29252/wjps.10.1.30

**Published:** 2021-01

**Authors:** Wael H. Mahmoud

**Affiliations:** 1 Department of Plastic and Reconstructive Surgery, Faculty of Medicine, Tanta University, Tanta, Egypt.

## Abstract

**BACKGROUND:**

We aimed to review the treatment and outcome of patients’ undergone reconstruction of large full thickness scalp defects with exposed calvarium after oncologic resection with the combined local flap and split-thickness skin graft (STSG) technique.

**METHODS:**

A retrospective review of 45 patients with scalp defects secondary to tumor extirpation was performed at the Plastic Surgery Department, Tanta University Hospital, Tanta, Egypt from Nov 2016 to Nov 2019. Patients, with large (>50 cm2) and full-thickness (exposed calvarium) scalp defects, who underwent scalp reconstruction with the combined local flap and STSG technique and had completed their medical records were enrolled.

**RESULTS:**

Only 38 met the inclusion criteria. Thirty-three were male (86.8). The mean age was 61.5 years. The lesions removed were BCC in 30 cases (78.9%) and SCC in 8 cases (21.1%). Defect sizes ranged from 55 to 196 cm2. There was complete survival of all flaps. Complications were noticed in 5 patients (13.2%);2 developed small hematomas, 2 suffered from partial graft losses and one had wound infection. The follow-up period ranged from 6 to 27 months. Overall, 34 patients were satisfied with the functional and cosmetic results (89.5%), while 4 female patients weren't satisfied with the esthetic results (10.5%).

**CONCLUSION:**

The combination of local flap and skin graft technique is highly reliable, easy to perform and safe single-stage reconstructive modality of large skull exposed scalp defects, providing durable coverage and favorable esthetic outcome.

## INTRODUCTION

The scalp represents the superior margin of the body; it extends anteriorly from the supraorbital margin to the external occipital protuberance and superior nuchal line posteriorly, and is divided into non-hair bearing (as the forehead) and hair-bearing regions^1^. Therefore, scalp defects can be easily caused by numerous etiological factors, including trauma, tumors, deep burns, congenital lesions, infections, or radiation therapy for either brain or skin tumors^2^. 

Tumors secondary to actinic damage have a constant rise in incidence, and the exposed position of the scalp leaves it as one of the primary sites for skin malignancy^3^. 

Reconstruction of scalp defects following tumor resection presents a complex clinical challenge for many reasons, including the rather excessive width and depth of these defects with potential calvarial exposure to ensure the oncologic radicality; the relative limited laxity of scalp tissues; the remoteness of the donor sites from the scalp^4^. Besides, associated co-morbidities especially among elderly patients that limit anesthetic tolerance. Another issue is the patients' expectations of cosmetic appearance^5^. There are many reconstructive modalities to be selected according to the size and depth of scalp defects, including primary closure^6^, tissue expansion^7^, local flaps^8^, skin grafts^9^, distant pedicled flaps^5^, or free flaps^1^. Nevertheless, each therapeutic option has its advantages and limitations. 

Large scalp defects with exposed calvarium require an organized approach for reconstruction. When a local scalp flap is harvested to cover the exposed bone to prevent calvarial desiccation and subsequent osteomyelitis, a considerable challenge may occur in repairing the flap's donor site, especially in patients with limited local soft tissue availability^10^. The flap's donor site or the secondary defect resulting from flap harvesting can be resurfaced by split-thickness skin graft (STSG) ^11^. This combined local flap and graft technique is used as a single operative procedure, avoiding multiple surgeries that may have significantly higher morbidities. Moreover, it is more cosmetically appealing due to replacement "like with like" tissue^12^. 

In this study, we aimed to describe our clinical experience in reconstruction of large full-thickness scalp defects with exposed calvarium after oncologic resection with the combined local flap and split-thickness skin graft (STSG) technique and discuss our results in terms of postoperative complications and outcomes.

## MATERIALS AND METHODS

In this retrospective study, we reviewed the treatment of 45 patients with scalp defects secondary to tumor resection. All scalp reconstructions were performed at our Plastic Surgery Department, Tanta University Hospital, Tanta, Egypt from Nov 2016 to Nov 2019. Patients with large (>50 cm^2^) and full-thickness (exposed calvarium) scalp defects, who underwent scalp reconstruction with the combined local flap and STSG technique and had completed their medical records were enrolled. Patients with history of prior scalp surgery or radiations were excluded from the study. The data gathered included patient demographics; co-morbid conditions; tumor types and localizations; size of defects; type of complications; patients' subjective satisfaction; functional and esthetic results evaluated by two independent plastic surgeons (i.e. poor, fair, good and excellent); and follow up period. 

After approval of our University Ethical Committee, and the written informed consents were taken, all cases were subjected to incision biopsy to detect the tumor pathology, and assessment of the cervical lymph nodes status and the presence of distant or loco-regional metastasis. 

**Surgical technique**

**Fig. 1:**
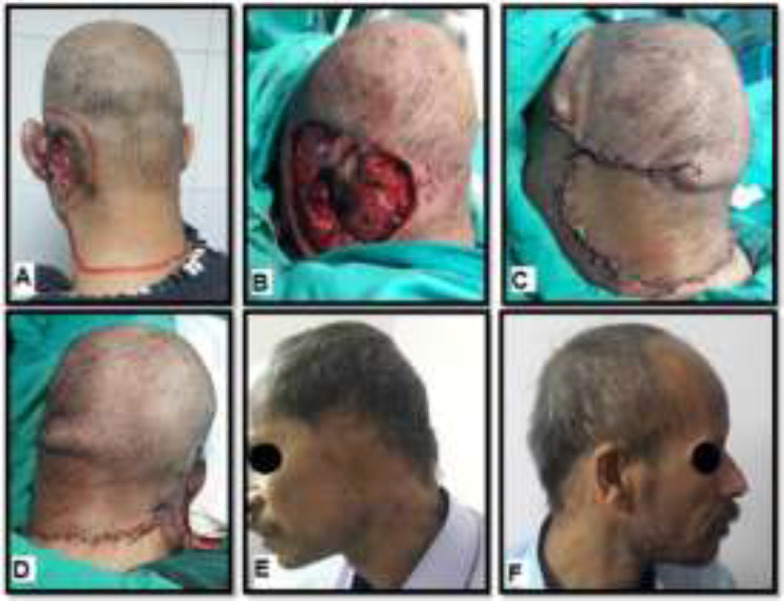
A 42-year old male with BCC in the left tempro-occiptal area, involving the ear. **(A).**Pre-operative marking of the flap and the adequate margins of the tumor. **(B).** Intra-operative view after the lesion was excised; the size of the defect was 140 cm^2^. **(C).**Intra-operative view shows flap harvesting and insetting. **(D).** Intra-operative view shows covering of the secondary defect by STSG. **(E, F).** Four months postoperative view shows good functional and esthetic outcome

All procedures were done under general anesthesia. The design of the flap and the adequate margins of the tumor were marked (Figure 1A). The area was infiltrated with adrenaline solution (1:500,000). The lesion was excised with at least 1cm margin of the surrounding tissue including the pericranium (Figure 1B). After obtaining free margins by frozen section, large scalp rotation flap (i.e. planned to be 2 to 3 times larger than the defect) was elevated by dissection at the subgaleal plane, paying particular attention to avoid injury to the underlying periosteum. Whenever there was a need for additional mobility of the flap, the flap's lateral incision was enlarged towards the flap base and the galea was scored at 1cm intervals in the flap movement direction, to achieve coverage of the primary surgical defect without tension. Thorough hemostasis was done with diathermy. The flap was secured in position using few interrupted sutures (Figure 1C). Rotation flaps often associated with dog ears, not excised immediately in order not to increase the risk of distal flap necrosis. The secondary surgical defect caused by the flap's donor site was covered by thick STSG harvested from the thigh (Figure 1D). Vacuum drains and compressive bandages were applied to all patients. Intravenous antibiotics were given for 48 h followed by five days of oral antibiotics. The dressing was removed on the 5^th^ postoperative day. Cases were seen at 10^th^ to 14^th^ postoperative days for suture removal, and also monthly for follow-up.

## RESULTS

Only 38 patients met the inclusion criteria and were enrolled in this study. Thirty-three patients were male (86.8%) and five patients were female (13.2%) with a mean age of 61.5 yr old (range: 42-75 yr old). Twenty-five patients remained active smokers (65.8%). Moreover, 17 patients had arterial hypertension (44.7%), 12 patients had type Π diabetes mellitus (31.6%), and 10 patients had ischemic heart diseases (26.3%). Thirty patients had Basal cell carcinoma (BCC) (78.9%) and eight patients had Squamous cell carcinoma (SCC) with no cervical lymph node affection or distant metastasis (21.1%). Defect sizes ranged from 55 to 196 cm^2^ (mean: 106.2 cm^2^). Locations of resultant scalp defects are presented in Table 1.

**Fig. 2:**
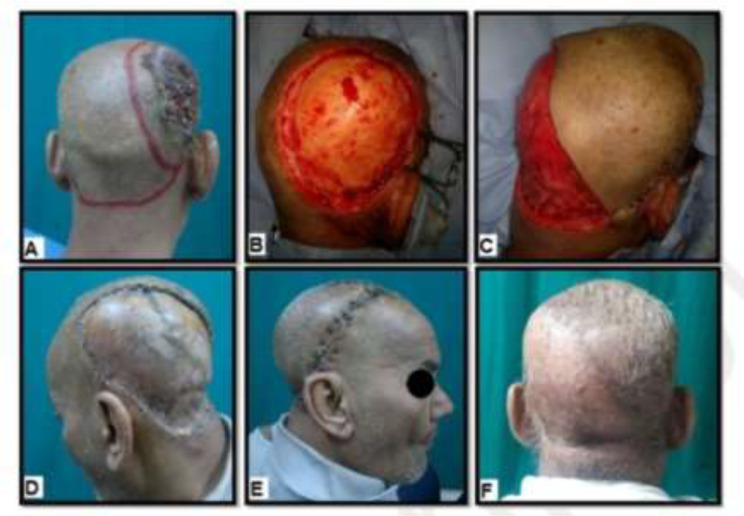
A 58-year old male with BCC in the right tempro-parietal area. **A)** Pre-operative marking of the flap and the adequate margins of the tumor. **B)** Intra-operative view after the lesion was excised; the size of the defect was 154 cm^2^. **C) **Intra-operative view shows flap harvesting and insetting.** D, E)** Postoperative view shows covering of the secondary defect by STSG and complete survival of the flap. **F)** Six months postoperative view shows good functional and esthetic outcome

**Fig. 3:**
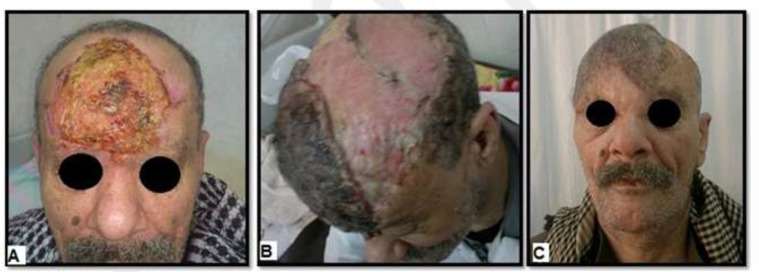
A 65-year old male with BCC in the forehead (frontal area). **A)** Pre-operative view. **B)** Postoperative view shows covering of the secondary defect by STSG. **C)** Three months postoperative view shows good functional and esthetic outcome

**Fig. 4:**
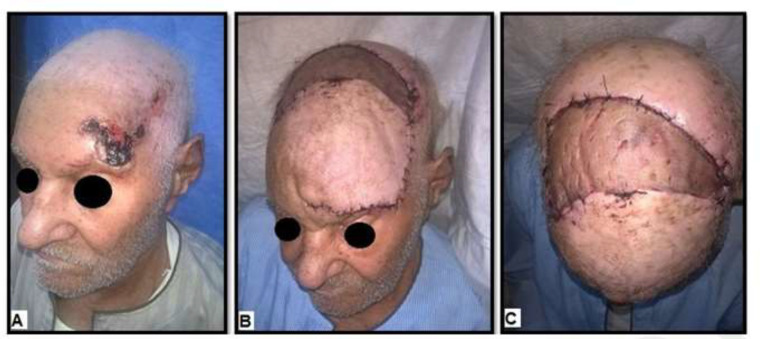
A 75-year old male with BCC in the left fronto-parieto-temporal area. **A)** Pre-operative view. **B)** Postoperative view shows complete survival of the flap**.** **C)** Postoperative view shows covering of the secondary defect by STSG

In general, there was complete survival of all flaps (Figure 2-4), there was no peri-operative mortality, and the postoperative course was uneventful in thirty-three patients (86.8%). Five patients had minor complications (13.2%), including small hematoma formation (about 50 cc in volume) in two hypertensive patients under medical treatment (5.3%), treated by bedside evacuation; partial graft loss in two patients (5.3%), attributed to periosteal injury during flap dissection, and they were treated by conservative dressing; and wound infection in one diabetic patient on insulin treatment (2.6%), treated by dressing and antibiotic based on culture and sensitivity. With regards to the surgical outcome as detected objectively by the two independent plastic surgeons, was excellent in six patients (15.8%), good in twenty-eight patients (73.7%), fair in three patients (7.9%), and poor in one patient (2.6%). Thirty-four patients were satisfied with the functional and cosmetic results of soft tissue coverage (89.5%), while four female patients were not satisfied with the esthetic results (10.5%).

**Table 1:** Patient data

**Age(yr)**	
Range Mean	42 -75 61.5
**Sex, n (%) **	
Male Female	33(86.8) 5 (13.2)
**Co-morbidities, n (%) **	
Diabetes mellitus Hypertension Ischemic heart disease Smoking	12 (31.6) 17 (44.7) 10 (26.3) 25 (65.8)
**Tumor type, n (%) **	
Basal cell carcinoma (BCC) Squamous cell carcinoma (SCC)	30 (78.9) 8 (21.1)
**Size of defect, cm** ^2^	
Range Mean	55 – 196 cm^2^ 106.2 cm^2^
**Location of defect, n (%) **	
Parietal Parieto-temporal Temporal Tempro-occiptal Occiptal Frontal Fronto-parieto-temporal	9 (23.7) 8 (21.1) 6 (15.8) 3 (7.9) 5 (13.2) 4 (10.5) 3 (7.9)

The follow-up period ranged from six to twenty-seven months (mean: 15.6 months). None of our cases had local recurrence or distant metastasis till the end of the follow-up period (Table 2). 

**Table 2:** Outcome data

**Complications, n (%) **	
Hematoma Partial graft loss Infection Overall complications	2 (5.3) 2 (5.3) 1 (2.6) 5 (13.2)
**Surgical outcome, n (%) **	
Excellent Good Fair Poor	6 (15.8) 28 (73.7) 3 (7.9) 1 (2.6)
**Patients' satisfaction, n (%) **	
Satisfied Dissatisfied	34 (89.5) 4 (10.5)
**Follow up, months**	
Range Mean	6 -27 15.6
**Tumor recurrence within follow up period, n (%)**	0 (0)

## DISCUSSION

The scalp is a common site for a variety of skin malignancies. Large full-thickness scalp defects caused by tumor extirpations often turn out to be a reconstructive challenge^13^. Although a diversity of reconstructive techniques have been advocated, successful outcome often mandates profound knowledge about the scalp vascularity and anatomy as well as the depth of the defects, especially with exposed calvarium. Nevertheless, in some patients associated co-morbid conditions may limit reconstructive options recommended elsewhere^14^. Primary closure remains the simplest method for hairy coverage. However, only patients with small defects (<3cm) approximated without significant tension are good candidates for primary closure^6^. The use of tissue expanders allows a larger area of hairy coverage but it is not free of morbidities and not recommended in acute injuries because of the risk of contamination and exposure of the implant^7^. 

Skin grafts can be used as a simple method for resurfacing large defects. They need a well-perfused wound bed as intact periosteum, even if there is insufficient pericranium through milling the outer table of the calvarium until fine bleeding occurs that allows grafting later on. Nonetheless, this technique exhibits inferior cosmetic results and less durability with possible trophic ulcers^9^. Distant pedicled flaps as the trapezius, pectoralis, or latissimus dorsi myocutaneous flaps have been described to provide durable and reliable coverage for large defects. However, their disadvantages remain the non-hairy bulky cover and the donor site morbidity^5^. Free tissue transfer offers stable coverage for large full-thickness scalp defects. Nevertheless, free flaps are somewhat technically demanding, time-consuming with more anesthetic exposure, and associated with possible donor and recipient sites morbidities^1^.

Local flaps can be used successfully for coverage of moderately sized scalp defects with hair-bearing tissue, especially with sufficient undermining. Yet it is not adequate in patients with large defects due to insufficient elasticity of scalp tissue^8^. In these instances, a large scalp rotation flap can be raised to transfer the defect to a less esthetically sensitive area with back-grafting of the donor site^15^.

In this study, we aimed to review the treatment and outcome of patients who undergone reconstruction of large full-thickness skull exposed scalp defects following oncologic resection, with the combined local flap and STSG technique.

We considered scalp defects (> 50cm^2^) to be large. In a similar study^5^, scalp defects bigger than 50 cm^2^ considered to be large, those more than 100 cm^2^ to very large, and those exceeding 200 cm^2^ to be extensively large. The authors noted that, for defects between 50 and 100 cm^2^, it is preferable to use large local flap and to graft, the donor site, while for defects larger than 100 cm^2^, and the best tool for reconstruction is free tissue transfer unless there is contraindication. In another study^2^, the scalp defects were classified into three groups as follows: 20-50 cm^2^ (large), 50-100 cm^2^ (very large), and >100 cm^2 ^(extremely large). Similarly, they managed to cover all defects with the combined local flap and STSG technique and considered this technique is a sufficiently good one for scalp reconstruction especially when the defects are large, with exposed calvarium.

In our series, scalp flaps were optimally designed to be significantly large to overcome the tough galea aponeurotica and the convex nature of the skull to cover the defect, incorporate a major vascular pedicle to maintain an axial blood supply to avoid the risk of necrosis, and preserve the native hairline as much as possible. All scalp reconstructions during 15 years were reviewed and found that local flaps with or without skin grafts are considered the workhorse for scalp reconstruction in their institution ^14^. This technique was associated with low complication rate (3.4%) when used to cover defects up to 150 cm^2^. The key to this approach is to harvest the flap large enough to cover the defect and minimize the number of flaps [i.e. single large flap or large double-opposing flaps (yin-yang)]. We noticed that dog-ears associated with rotation flaps uniformly flatten with time and needed no further correction. Even other studies ^16^^-^^18^, as well agree; the “dog ear” plasty is barely needed. If required, delayed excision has to be performed after some time in an office setting without any difficulty.

Our study demonstrated a low complication rate (13.2%) and good outcome (73.7%) of patients. These findings agreed with Aldabagh et al.^12^, who found that the combined flap and graft technique was the preferred method for reconstruction of moderate to large scalp defects with exposed bone due to its operative ease, low complication rate (10%), durability, and good esthetic results in all patients. Unlike us, a higher complication rate (26.3%) (i.e. 1 hematoma, 1 wound dehiscence, 1 delayed wound healing, 1 wound infection, and 1 distal flap necrosis) was attributed to inclusion of patients with prior scalp surgery, cryotherapy and radiation therapy ^4^. When the defects are more than 50 cm^2^ on the forehead and exceeding 200 cm^2^ on the scalp, a free tissue transfer should be regarded.

89.5% of our patients had satisfactory functional and cosmetic results, while 10.5% of the patients weren't satisfied with the esthetic results. They were women and they did not require any further surgical interference. Three patients underwent the combined flap and graft technique and weren't satisfied with the functional and cosmetic results and one of them developed an unstable scar at the graft site and required a subsequent surgery^3^. In another six patients, they performed the Orticochea technique in which 2 flaps based on the superficial temporal vessels were used to reconstruct the 1ry defect and on large occipital flap was used to cover the 2^nd^ ry defect, and the patients were satisfied with the results. Six patients weren't satisfied with the cosmetic results and tissue expander procedures were used to regain hairy areas later on ^19^. The combined local flap and graft technique is best applied in hair-bearing areas, while free tissue transfer is preferred in non-hair bearing regions.

In an algorithmic approach to reconstruct acquired scalp defects, most scalp defects can be resurfaced with local flaps with or without skin grafts ^10^. If local tissues are inadequate, tissue expansion is of choice, as it replaces damaged scalp with like tissue. If inadequate scalp tissue is present to allow expansion, free flaps are good choice, but always consider patient co-morbidities in performing reconstructions. 

The current study was limited by the retrospective nature of the data available. Moreover, the numbers of patients were relatively small. Considerably, more prospective trials on larger sample size and for a longer follow-up interval would help us to establish a greater degree of accuracy about the ideal method for oncologic scalp reconstruction in the multimorbid aged patients.

## CONCLUSION

The combination of local flap and skin graft technique is highly reliable, easy to perform and safe single-stage reconstructive modality of large skull exposed scalp defects, providing durable coverage and favorable esthetic outcome.
